# Inhibition of the mevalonate pathway affects epigenetic regulation in cancer cells

**DOI:** 10.1016/j.cancergen.2015.03.008

**Published:** 2015-05

**Authors:** Heidrun Karlic, Roman Thaler, Christopher Gerner, Thomas Grunt, Katharina Proestling, Florian Haider, Franz Varga

**Affiliations:** a Ludwig Boltzmann Cluster Oncology, Vienna, Austria; b Ludwig Boltzmann Institute of Osteology at the Hanusch Hospital of Social Health Insurance Vienna (WGKK) and Austrian Social Insurance for Occupational Risks (AUVA) Trauma Centre Meidling, 1st Medical Department, Hanusch Hospital, Vienna, Austria; c Institute of Analytical Chemistry, Faculty of Chemistry, University of Vienna, Vienna, Austria; d Signaling Networks Program, Division of Oncology, Department of Medicine I, Medical University Vienna, Vienna, Austria; e Comprehensive Cancer Center, Medical University Vienna, Vienna, Austria; f Department of Obstetrics and Gynecology, Medical University of Vienna, Vienna, Austria

**Keywords:** Mevalonate pathway, statins, bisphosphonates, epigenetics, cancer metabolism

## Abstract

The mevalonate pathway provides metabolites for post-translational modifications such as farnesylation, which are critical for the activity of RAS downstream signaling. Subsequently occurring regulatory processes can induce an aberrant stimulation of DNA methyltransferase (*DNMT1*) as well as changes in histone deacetylases (HDACs) and microRNAs in many cancer cell lines. Inhibitors of the mevalonate pathway are increasingly recognized as anticancer drugs. Extensive evidence indicates an intense cross-talk between signaling pathways, which affect growth, differentiation, and apoptosis either directly or indirectly via epigenetic mechanisms. Herein, we show data obtained by novel transcriptomic and corresponding methylomic or proteomic analyses from cell lines treated with pharmacologic doses of respective inhibitors (i.e., simvastatin, ibandronate). Metabolic pathways and their epigenetic consequences appear to be affected by a changed concentration of NADPH. Moreover, since the mevalonate metabolism is part of a signaling network, including vitamin D metabolism or fatty acid synthesis, the epigenetic activity of associated pathways is also presented. This emphasizes the far-reaching epigenetic impact of metabolic therapies on cancer cells and provides some explanation for clinical observations, which indicate the anticancer activity of statins and bisphosphonates.

For more than 100 years, it has been known that cholesterol may accumulate in cancerous tissues (1) and plays a critical role in cancer progression, thus emphasizing the therapeutic potential of lowering cholesterol and downregulating the mevalonate pathway in cancer prevention and treatment (2). The mevalonate pathway converts acetyl-coenzyme A (acetyl-CoA) to isoprenoids, thus supplying key metabolites for cholesterol and steroid synthesis. It comprises a series of enzymatic reactions that occur in the endoplasmic reticulum. The rate-limiting step is catalyzed by 3-hydroxy-3-methylglutaryl-coenzyme A (HMG-CoA) reductase, which converts HMG-CoA to mevalonate. This reaction is inhibited by statins, whereas bisphosphonates target more downstream reactions in this pathway, such as farnesylation and geranylgeranylation.

Meanwhile, there exists an increasing amount of data, which indicate that statins, as well as bisphosphonates, target the three most important epigenetic levels: DNA methylation, histone deacetylation, and microRNAs (Figure 1).

The best-described epigenetic roles of statins and bisphosphonates result from a reduction of the membrane anchoring from RAS and associated signaling toward DNA demethylation (3,4), or downregulation of the histone deacetylase HDAC2 via the RAS/PI3K/mTOR pathway (5) in addition to a direct competitive inhibition of HDAC2 by statins (6). Reduction of homocysteine, which is produced in the one carbon metabolism (OCM), also leads to a downregulation of the DNA methyltransferase DNMT1 (7) and a shift in the NAD(P)^+^/NAD(P)H-ratio toward NADP, with apparent consequences for histone modifications (8–10) and DNA repair through breakdown of poly-ADP-ribose (9). The downregulation of geranylgeranylation of another small GTPase, RHOA, and associated signaling (11) downregulates HDAC1 (12) and promotes vitamin D−associated epigenetic effects (13–15) by preventing CYP24A1-induced degradation of vitamin D3 (16–18).

In this study, simvastatin was chosen as a representative statin for transcriptomic studies, because a large-scale investigation was already performed with this drug and it was the first statin drug used extensively in clinical practice for control of elevated cholesterol. Epigenetic studies with simvastatin emphasize its role as a direct inhibitor of HDAC1 and HDAC2 (6) or as an inducer of respective microRNAs (19–21). Ibandronate was selected as a representative bisphosphonate, because it is already known for its epigenetic impact (3).

## Materials and methods

### Cell cultivation treatment and NADP^+^/NADPH analyses

Cells were cultivated in cell culture flasks at 37°C and 5% CO_2_. The culture media were as recommended by the American Type Culture Collection (ATCC) for MDA-MB-231 breast cancer DMEM (Sigma-Aldrich, St. Louis, MO, USA), which contained 10% fetal calf serum (FCS); PC-3 prostate carcinoma DMEM-F12 (Sigma-Aldrich) with 10% FCS. MG-63 and U2-OS osteosarcoma were cultured in AlphaMEM (Biochrom, Berlin, Germany) medium containing 10% FBS. For the HMC1.1 cell line, we used Iscove's Modified Dulbecco's Medium (IMDM; Thermo Fisher Scientific, Waltham, MA) supplemented with 260 nM thioglycerol (Sigma-Aldrich) and 20% fetal bovine serum (FBS). All culture media contained 10 μg/mL gentamycin (Sigma-Aldrich). To guarantee optimal growth, cells were split two times a week and reseeded at a density of 2−5 × 10^5^ cells/mL.

One day after splitting, 32 μM simvastatin (Sigma-Aldrich) or 150 μM ibandronate (Sigma-Aldrich) were added to the culture medium for 72 hours. This is the dose that attenuated cell proliferation with a half maximal effect (EC50) (data not shown).

NADP/NADPH analyses were performed directly in 96-well culture plates after 24 or 48 hours, according to the manufacturers' instructions of the NADP/NADPH Glo Assay (Promega, Madison, WI, USA).

### Gene expression analysis

For comparative analysis of selected genes, we synthesized cDNA with the First Strand cDNA Synthesis Kit as described by the supplier (Roche, Rotkreuz, Risch, Switzerland). The obtained cDNA was subjected to PCR amplification with a real-time thermal cycler (Corbett Research; Fisher Scientific, Schwerte, Germany). FAM-labeled TaqMan gene expression probes and primers sets (all from Applied Biosystems, Foster City, CA) were used according to the conditions suggested by the suppliers. For normalization of expression, we used VIC-labeled *GAPDH* and *18S* TaqMan probes and primers sets in the same reaction vial (*GAPDH* 4310884E, *18S* 4319413E; Applied Biosystems). Quantification of mRNA expression within the samples was examined using the comparative Ct method (22).

### Transcriptomics and proteomics analysis

Analysis and data evaluation for the Affymetrix arrays (Type Human Gene 1.0 ST Array; Affymetrix, Santa Clara, CA, USA) were commercially obtained from an internationally certified institution (Kompetenzzentrum für Biofluoreszenz, Regensburg, Germany). PathVisio software (23) was applied for specific analyses of defined pathways from Affymetrix arrays (Type Human Gene 1.0 ST Array). Proteomics analyses were conducted as described (24,25).

## Results

### Downregulation of DNA methyltransferase

**Figure 1:**
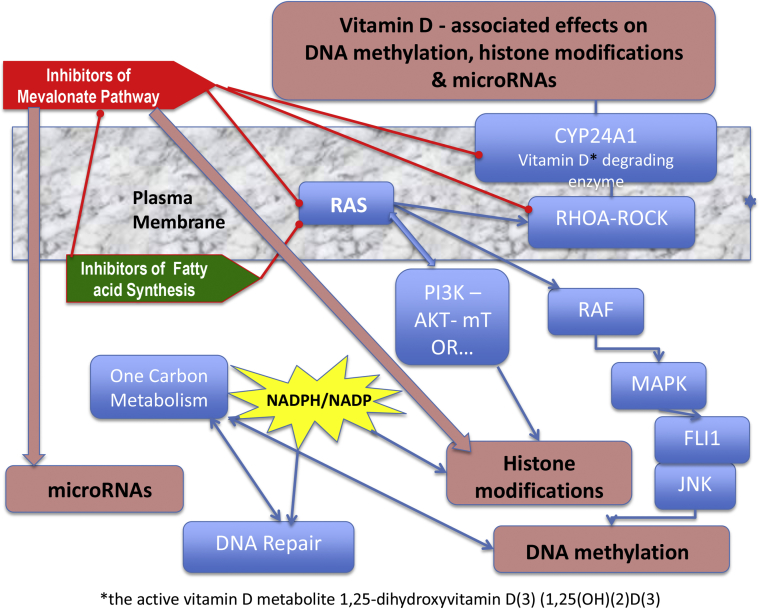
Inhibition of the mevalonate pathway influences the stability of the plasma membrane. It inhibits isoprenylation of the small GTP-binding proteins and, therefore, the activity of RAS signaling. As a consequence, RAS signals via RAF into the MAPK pathway, an inhibited signaling via FLI1 and JNK (c-JUN N-terminal kinase), leads to a downregulation of DNMT1. The cross-talk of RAS with PI3K-AKT-mTOR signaling influences the expression of HDACs. Additional metabolic pathways influenced by RAS signaling are glucose uptake and the OCM, which may both be fueled by activating mutations of the P53 gene (*TP53*) and play essential roles in DNA repair and inflammation. Similar to the inhibition of HMG-Co-A reductase, a downregulation of these pathways changes the concentration of NADPH. In addition, there is also a downregulation of the RHOA-ROCK signaling and the associated vitamin D degrading enzyme CYP24A1 (18). This could induce a series of vitamin D−associated effects on fatty acid metabolism and epigenetics, for example (13).

**Figure 2:**
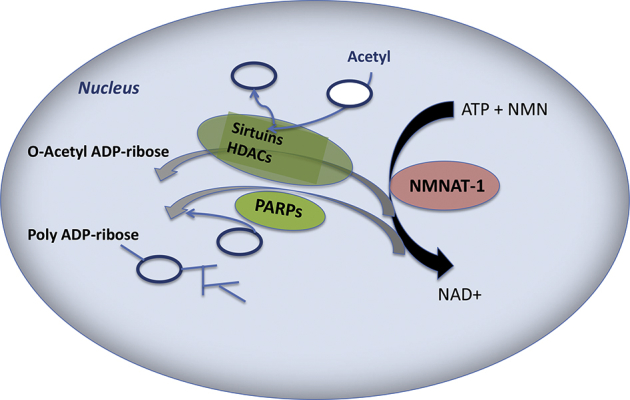
NAD(P)^+^ biosynthesis and major NAD(P)^+^-mediated signaling pathways affect histone (de)acetylation (modified according to (36)). Simvastatin and ibandronate induce upregulation of the *NMNAT* (nicotineamide mononucleotide acetyltransferase), which synthesizes NAD from ATP and NMN (nicotineamide mononucleotide). NAD^+^-consuming reactions from PARP (polyADP ribose polymerase), HDACs, and sirtuins are downregulated by inhibitors of mevalonate synthesis in cancer cells.

Blocking the mevalonate pathway inhibits isoprenylation of the small GTP-binding proteins and, therefore, the activity of signaling from GTP-binding proteins such as RAS. RAS signals via RAF into the MAPK pathway (26,27). Consequently, the whole cascade is affected and associated *DNMT1* expression (4,28) is downregulated (Figures 1 and 2, Table 1) (4).

Such data could explain far-reaching consequences, including demethylation and activation of key mediators of apoptosis (3) and differentiation (29), and would have a major impact on metabolism (30). In addition, upregulation of DNMTs in malignancies may be driven by HDAC2 (31). Thus, a drug-induced downregulation of these enzymes underscores the anticancer activity of statins and bisphosphonates.

### Downregulation of HDACs

Our transcriptomic analyses indicated a downregulation of histone deacetylases (Table 2).

The expression of HDACs is influenced not only by the cross-talk of RAS with PI3K-AKT-mTOR signaling (32) (Figure 1 and Table 2) (4), but also by metabolites such as NAD(P)^+^ and NAD(P)H, which are also targeted by metabolic modifiers such as statins. Besides the “classical” NAD-dependent histone deacetylases from the SIRT family, HDAC1 and HDAC2 are also regulated by this metabolite (33), as shown in Figure 1 (4) and Table 2.

### Regulation of microRNAs

The mean percentage of significantly downregulated microRNAs in a total of 1,199 microRNAs, which were detectable in our gene chips, was 14.8% in simvastatin-treated and 14.2% in ibandronate-treated cell lines. MicroRNA-34a, which regulates the NAD^+^-dependent histone deacetylase SIRT1 as well, as HDAC1 and HDAC7 (2,34), was downregulated with simvastatin in all cancer cell lines investigated in this study, but most significantly in simvastatin-treated MDA-MD-231 cells (Table 3).

The mean percentage of significantly upregulated microRNAs in a total of 1,199 microRNAs, which were detectable in our gene chips, was 21.9% in simvastatin-treated and 14.4 % in ibandronate-treated cell lines. The most significantly upregulated microRNA in simvastatin-treated MDA-MB 231 cells was microRNA-612, which is known to reduce stemness and to attenuate resistance against 5-fluorouracil in cancer cells (35). MicroRNA-612 was also significantly upregulated in simvastatin-treated PC-3 cells as well as in MG-63 and HMC-cells, which had been treated with simvastatin (Table 3).

### Epigenetic impact of OCM

**Figure 3:**
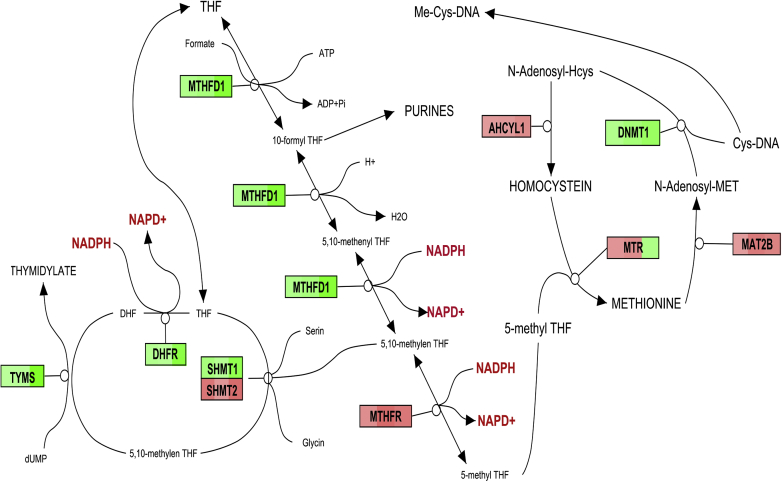
Results from a transcriptomic analysis of the OCM: Downregulated genes were, dependening on their level colored in green, upregulated in red. The analyzed cell lines are from the left to the right: U2OS osteosarcoma treated with ibandronate and simvastatin; PC-3 prostate cancer cells treated with ibandronate. The labels are the actual gene names according to the NCBI gene database. *Abbreviations: MTHFD*, methylenetetrahydrofolate dehydrogenase; *MTHFR*, methylenetetrahydrofolate reductase; *DHFR*, dihydrofolate reductase; *TYMS*, thymidylate synthetase; SHMT, serine hydroxymethyltransferase; *AHCYL*1, adenosylhomocysteinase-like 1; *MTR*, 5-methyltetrahydrofolate-homocysteine methyltransferase; *DNMT1*, DNA (cytosine-5-)-methyltransferase 1; *MAT2B*, methionine adenosyltransferase II, beta.

Data from our genome-wide expression analysis indicated that the majority of transcribed genes from folate metabolism, which is also known as OCM, was downregulated by simvastatin in HMC 1.1, U2-OS, and MDA-231 cells at the mRNA level (Figure 3). Proteomic data from U2-OS also confirmed this at the protein level.

The “starter” molecule of OCM dihydrofolate reductase (DHFR) is the target for many anticancer and antibiotic therapies, including methotrexate and trimethoprim. In our study, DHFR was downregulated in the U2-OS proteomic assay: −8% by ibandronate and −49% by simvastatin. OCM is also known to provide key metabolites, such as NAD(P)^+^, which are important cofactors for histone-modifying enzymes (33,36,37).

Downregulation of the enzymes that are important for synthesis of polyglutamate suggests a lowering of the pool of metabolites that are important for folate synthesis, in addition to an increased NADP^+^/NADPH ratio in responsive cell lines, such as MDA-MB-231, but not in less aggressive cells such as MG-63. This could provide an explanation for previous observations indicating that a combined treatment of Ehrlich carcinoma cells with the cholesterol-lowering drug atorvastatin showed an additive effect with methotrexate on tumor tissue volume and of the apoptotic index (38). An explanation for this finding was the downregulation of most enzymes of the OCM by simvastatin, as shown in Figure 3, in three tumor cell lines that may act additionally to the inhibition of the DHFR by methotrexate (Table 4), however, it is not clear which of these pathways is responsible for the antiproliferative effect of statins.

However, this could also be due to a lack of NADPH, because the following NADPH-producing reactions are downregulated by statins: 1) glycolysis, including the NADPH-producing pentose-phosphate cycle as well as the KREBS or tricarbonic acid cycle (39), 2) the fatty acid oxidation (40), 3) the OCM, which was identified as a major producer of NADPH by quantitative flux analysis (41), and 4) *TYMS*, which is among the most downregulated genes analyzed in our transcriptomic study, is known to convert dUMP to dTMP in the presence of NADPH and serine (42). Thus, a lack of NADPH could be responsible for the downregulation of this gene in responsive cell lines (Table 4).

The link to epigenetics is emphasized by data indicating that NADPH can stimulate class I HDAC activity in vitro and in vivo (33). This affects regulation of the protein poly (ADP-ribose) polymerase (PARP) and associated effects on chromatin relaxation and DNA repair (43). Thus, a lack of NADPH could explain the observed downregulation of HDACs and the observed downregulation of DNA repair factors by simvastatin (Figure 2).

Inhibitors of the HMGCR (44,45) and DHFR enzymes are known for their anti-inflammatory activities (46), which are related to their antioxidant properties resulting from inhibition of NADPH-dehydrogenases. By our proteomic analysis of simvastatin-treated U2OS NADH-dehydrogenases, NDUFA8 (−4%), NDUFV2 (−8%), and NDUFS2 (−20%) were downregulated, in addition to the NADH cytochrome B5 reductase CYB5R1 (−34%) and the NAD^+^-dependent HDAC, SIRT1 (−3.2%).

Inhibition of the OCM downregulates the production of homocysteine (Hcys) (see Figure 3) (47). Hcys promotes the production of serum amyloid A (SAA) (48), and this is associated with a stimulation of inflammatory interleukins (49).

Hcys modulates expression of osteoblastic genes, but most important, it downregulates procollagen-lysine-1,2-oxoglutarate-5-dioxygenases as well as lysyl oxidase (*LOX*). The downregulation of both genes, which are involved in collagen cross-linking could contribute to decreased bone matrix quality. We have shown that the downregulation of *LOX* is mediated by Hcys via interleukin-6 (IL6), Friend leukemia integration 1 (FLI1), and *DNMT1* and epigenetically regulated via promoter methylation (7). This has a relevance to inflammation-associated osteopenia, which is associated with a downregulation of *LOX* in response to tumor necrosis factor alpha (TNFα) (50). Pathologically inhibited LOX is upregulated by statins via inhibition of geranylgeranylated proteins, such as Rho-kinases (51), as well as by promoter demethylation (based on our own data). Table 5 demonstrates that the bisphosphonate ibandronate upregulated LOX as well. Recent findings suggest a similar mechanism for *LOX* (7) by demonstrating that the bisphosphonates upregulate *FAS* via promoter demethylation.

### Regulation of vitamin D metabolism by inhibitors of mevalonic acid metabolism may also affect epigenetic mediators

As shown in Figure 1, inhibitors of the mevalonic acid pathway have the potential to upregulate vitamin D metabolism through attenuation of a vitamin D degrading enzyme.

In addition, inhibitors of fatty acid synthase (FASN), such as C75, may also target the mevalonic acid pathway (52) and RAS activity. In our study, FASN was downregulated by inhibitors of the mevalonic acid pathway, which confirmed previous studies (53). As shown in Table 6, FASN was downregulated by inhibitors of the mevalonic acid pathway.

**Figure 4:**
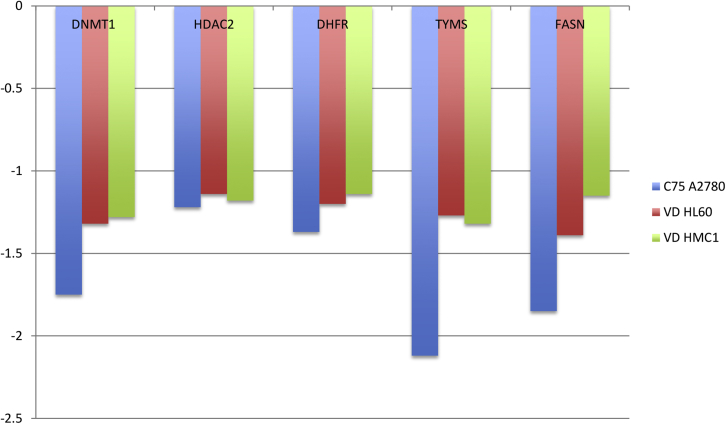
Effect of a FASN inhibitor (C75) or vitamin D3 on epigenetic regulators (*DNMT1* and *HDAC2*, key enzymes of OCM *DHFR* and *TYMS*, as well as *FASN*.

Furthermore, the association between FASN downregulation and PI3K signaling, which is already documented (54–57), could be confirmed by our data from respective genome-wide expression analyses (Figure 4), which included the FASN inhibitor C75 as a control, thus emphasizing this pathway as a potential target for anticancer therapy (58).

FASN inhibitors cerulenin and C75 induce cell cycle arrest and apoptosis in tumor cells. This is associated with elevation of CDKN1A (P21) (59), and similar effects were also observed upon treatment with statins (6) or 1,25-dihydroxy vitamin D3 (VD) (60). Interestingly, some authors mentioned that statins might induce an increase of VD (61), which appears to be a paradox, because VD synthesis depends on metabolites from the mevalonic acid pathway. However, it appears possible that statin-induced upregulation of the ATP-binding cassette transporter (62), which is also responsible for VD uptake, (63) could explain this phenomenon, in addition to the statin-induced downregulation of the vitamin D degrading enzyme CYP24A1 (17). Although speculative, it could also be possible that an additional pathway for isoprenoid synthesis, which is characteristic for bacteria (64), might have been “imported” by endosymbionts via phagocytosis.

A further comparative evaluation of our genome-wide expression analyses shows that drugs that downregulate FASN also downregulate OCM and vice versa (Figure 4).

## Discussion

### Downregulation of DNA methyltransferase

Our data (Table 1) confirm that statins exhibit demethylating properties. Inhibition of DNMTs can be seen even at low statin concentrations (0.25 μM), which are comparable to the serum levels of approximately 0.1 μM measured in patients treated with standard doses for hypercholesterolemia and far lower than the maximum safely achievable levels in humans. These low concentrations of statins are safe and well tolerated by patients for years. Thus, in contrast with many known DNMT inhibitors, statins downregulate DNMTs and induce DNA demethylation at nontoxic doses (29).

### Downregulation of HDACs

Our transcriptomic studies indicated a downregulation of at least five HDACs (Table 2). Our results were similar to previous reports, which indicated that statins and bisphosphonates act synergistically with HDAC inhibitors (65) and exert a direct competitive inhibition of HDAC2 (6), leading to an increased histone-H3 acetylation on the *SP1* sites of the promoter from *CDKN1A* (also known as P21).

Another study indicated that inhibiting *HDAC5* originated from a downregulation of the histone methylase *EZH2* (66), which was downregulated with both simvastatin and ibandronate in our study.

Such modifications may stimulate expression of *CDKN1A*, which is responsible for cell cycle arrest. A statin-induced cell cycle arrest and an accumulation of *CDKN1A* were shown in lymphoma cells (67). Thus, inhibition of isoprenoid synthesis by statins could explain data indicating that these drugs inhibit progression of epigenetically influenced diseases such as cancer (68) and hematologic malignancies, as shown in a survey of 578,000 adults (69).

### Regulation of microRNAs

Small non-coding RNAs (microRNAs) play an important role in the post-transcriptional regulation of a number of genes and their involvement in many pathological states, including the metabolic syndrome and cancer (70). Statins were shown to stimulate microRNA-33b (*MIR-33b*), which is known to repress *MYC*, thus inducing a cell-cycle arrest in G1 (21).

MicroRNA-33 is an intronic microRNA located within the sterol regulatory element-binding protein (*SREBP*) genes, which are one of the master regulators of cholesterol and fatty acid metabolism. Furthermore, this microRNA regulates the inflammatory cytokine production via cholesterol sensing in macrophages (19).

Furthermore, statins are also known to affect expression of microRNA-34a (Table 3), which regulates the NAD^+^-dependent histone deacetylase *SIRT1* (20) as well as *HDAC1* and *HDAC7* (34). Simvastatin was also shown to decrease microRNA-155 expression by interfering with the mevalonate-geranylgeranyl-pyrophosphate-RhoA signaling pathway (71).

Another target for microRNAs is the previously mentioned *TYMS*, which was most significantly downregulated by simvastatin or ibandronate in our study. The 3′ untranslated region of *TYMS* has predicted binding sites for several microRNA families, and altered expression of several microRNAs has been reported in ovarian carcinoma (both serous and unspecified type) when compared with normal tissue. Predicted microRNA target sites at *TYMS* also contain at least two polymorphisms (72). The *MIR-34a* is a critical microRNA, which is responsible for DNA damage, because it targets the TYMS gene (73) and also attacks glycolysis (74).

The most significantly upregulated microRNA in the simvastatin-treated MDA-MB 231 cells from our study was microRNA-612 (Table 3), which is known to reduce stemness and to relieve drug resistance to cisplatin and 5-fluorouracil, possibly by targeting *TYMS* in cancer cells (35). MicroRNA-612 was also significantly upregulated in simvastatin-treated PC-3 cells as well as in MG-63 and HMC-cells, which had been treated with simvastatin (Table 4).

In addition, it appears possible that statin-associated accumulation of Vitamin D might induce a larger number of microRNAs (15).

### Epigenetic impact of targeted metabolic pathways

Results from our transcriptomics analyses (Figure 3) demonstrated that statin-mediated downregulation of OCM could inhibit DNA synthesis, repair, and methylation directly (75) and indirectly via epigenetic activation of demethylated genes as well as associated microRNAs (70).

Evidence exists that other epigenetically active compounds, such as epigallocatechin-3-gallate (EGCG) that shares with statins the potential to downregulate mevalonate metabolism (76) as well as demethylating activity (77), also act on folate metabolism (78,79).

A tight linkage of the *DHFR* and *HMGCR* genes exists on chromosome 5q13.3-q14, and both genes are co-amplified in cell lines (such as K562), which are resistant to the DHFR inhibitor methotrexate (80). However, concerning gene regulation, mRNA of *HMGCR* was not significantly regulated with simvastatin or ibandronate, but a pronounced downregulation was observed for the *DHFR* and *TYMS* genes (Table 4). In four of five investigated cell lines (MDA-MB-231, MG-63, U2-OS, and HMC 1.1), both *DHFR* and *TYMS* were downregulated, thus emphasizing the close metabolic association of these two genes and their immediate impact on the two principal epigenetic regulators, *DNMT1* and *HDAC2* (Tables 1 and 2).

Methylene THF is also a cofactor of TYMS, which converts dUMP to dTMP by adding a methyl group. If cellular folate levels are low, uracil misincorporation occurs, leading to DNA strand breaks. Thus, the TYMS enzyme has been of interest as a target for cancer chemotherapeutic agents. It is considered to be the primary site of action for 5-fluorouracil, 5-fluoro-2-prime-deoxyuridine, and some folate analogs. On the protein level, we detected a −24% downregulation of TYMS with simvastatin (but no regulation of TYMS with ibandronate, despite a significant downregulation of mRNA) in U2-OS cells. This could confirm previous studies, which indicate a possible stimulatory effect on cancer cells resulting from folic acid fortification (81,82), which increases TYMS activity (83–86). However, statin-mediated downregulation of DNA repair may also result from a direct inhibition of oncogenic RAS (87).

HMGCR reduces HMG-CoA to mevalonic acid. Cancer-associated metabolic changes may shift the NA(P)D^+^/NAD(P)H ratio toward NADPH, which is similar to alcohol metabolism (88). Histone deacetylase activity is also NADP^+^-dependent; therefore, this could explain the similarity of targeted pathways, such as cholesterol synthesis, which is downregulated both by HDAC inhibitors (10) and simvastatin. Possibly, the downregulation of TYMS by the HDAC inhibitor vorinostat (89) could refer to downregulation of the same pathway. The concordant downregulation of the *DNMT1* and *HDAC2* genes could indicate some similarities in the action from inhibitors of DNMT1 and HDACs.

The downregulation of the OCM metabolite HCys and an associated epigenetically mediated stimulation of *LOX* (Table 5) could explain the beneficial effects of statins (90) and bisphosphonates in osteoporosis (91). Furthermore, it has been demonstrated that the propeptide of LOX (ppLOX), which is the liberated form of the LOX precursor by cleavage with protease bone morphogenetic protein 1 (BMP1), inhibits cancer-associated DNA repair (92). Downregulation of *TYMS* by both ibandronate and simvastatin in some of the tested cell lines from our own study could explain a previous study that demonstrated the effect of mevalonate pathway inhibitors on DNA damage response in human sarcoma cells (93).

Targeting of the key epigenetic enzymes and key enzymes from OCM was also observed by treatment of the FASN responsive cell line A2780 with the FASN inhibitor C75 or in the VD-responsive cell lines HL-60 and HMC-1.1 with VD, which is well documented for its epigenetic impact (14).

However, the attenuation of downstream regulators of FASN, as expected from treatment with C75 in the responsive A2780 ovarian cancer cell line, was just partially observed in simvastatin-treated cell lines (Figure 4). Recent data indicating a specific protective role of statins (94) against ovarian cancer underscore the possible impact of metabolic therapies in this disease. The use of A2780 cells as a model for the anticancer effect of simvastatin has been documented (95).

Downstream signaling of FASN affects the RAS-PIK3 kinase-AKT-mTOR pathways (54) and the associated role of this pathway for HDAC inhibition (96), with the latter also affected by treatment with statins and bisphosphonates (3,97). Another study explained the association of the RAS-PI3K-mTOR pathway to HDAC inhibition (5).

This emphasizes both the complex network of metabolic pathways, which influence epigenetic reactions, and the multiple activities from inhibitors of the mevalonic acid pathway.

## Conclusion and clinical perspectives

Our research provides some models for epigenetic mechanisms, which could explain many clinical studies that evaluated the association between statin use and a lowered cancer occurrence. Although populations encompassing more than 1 million men have been screened, there is also some controversy regarding the effectiveness of statins in preventing prostate cancer (98,99). However, a direct effect of statins on cultured cancer cells, including G1 cell cycle arrest, autophagy, and proteolytic degradation of steroid receptors, is well documented (100,101). Breast cancer cells treated in culture and in vivo as xenografts with lovastatin had reduced proliferative ability, which further decreased in the presence of *BRCA1* overexpression via regulation of the cell-cycle regulatory proteins cyclin D1-CDK4-p21WAF1/CIP1 (102).

Critical evaluations (103) also discuss studies postulating that statins could be a cancer-promoting class of drug, and, indeed, the observed changes in downregulation of enzymes that are associated with DNA repair or nucleotide synthesis, for example, could be pro-cancerogenic when they occur in normal cells.

However, the majority of studies indicates an inverse correlation between statin use and cancer risk in the sense of reduced cancer-related mortality among statin users (68). More important, further clinical trials are under way, with the primary end point of assessing the clinical utility of statins as prevention measures for cancer (103).

Targeting of the mevalonic acid pathway represents an example for a metabolic focus of targeting (potential) malignancies and their (micro)environments. This is a prerequisite for treatment assays as well as for future approaches for personalized therapies.

Currently, bisphosphonate-based drugs are used to treat bone diseases, including osteoporosis, tumor-induced hypercalcemia, and osteolytic cancer metastases (104). In addition to skeletal benefits, clinical studies have shown that bisphosphonates can suppress the proliferation of cancer cells, including prostate (105), breast (106), and colorectal cancers (107), as well as glioblastoma (108) and multiple myeloma (MM) (109). Further investigations have also provided evidence that some bisphosphonates improve the survival of patients with MM via mechanisms that may be both related as well as unrelated to the skeletal benefits (110,111). Similar results have been reported for patients with premenopausal breast cancer (112), although these findings seem to be more controversial (113).

In addition, the incidence of various types of inducible breast cancers in rats and mice could be reduced by feeding with statins, a process prevented by adding mevalonate, but not by adding farnesyl pyrophosphate. In rodent models of breast cancer, rats fed simvastatin had a lower incidence of induced mammary tumors (114). Regarding the mechanism of action, a significant mammary antitumor effect in mice via decreased p-MEK1 and/or MEK2 protein levels was detected, which act in the RAS/RAF/MEK/ERK cascade that drives cell proliferation (115), in addition to a proapoptotic shift in the Bcl-2/Bax protein ratio (116).

Animal models have indicated the antiproliferative effects of statins on lung, liver, colorectal (with evidence of epigenetic reprogramming), melanoma, medulloblastoma, ovarian, and prostate tumors (21,29,117–122).

Although the role of epigenetic markers in this scenario remains to be confirmed in larger data sets, it appears clear that the far-reaching epigenetic consequences from inhibitors of the mevalonic acid pathway are worth observing and investigating in detail.

## Figures and Tables

**Table 1:** Effect of ibandronate and simvastatin on the key epigenetic regulator *DNMT1*^a^

Gene expression	U2-Ibn	MG-Ibn	PC-Ibn	MDA-Ibn	U2-Sim	MG-Sim	PC-Sim	MDA-Sim	A2780-C75
*DNMT1* basal expression	10.0	11.3	10.8	11.3	10.0	11.3	11.6	11.3	9.6
*DNMT1* treated expression	9.7	11.3	10.0	11.3	9.5	11.3	10.3	10.0	8.8
***DNMT1* fold expression**	**−1.21∗**	**1.02†**	**−1.73^∗^**	**1.00**	**−1.47^∗^**	**1.06†**	**−2.47^∗^**	**−2.56^∗^**	**−1.75^∗^**

*Abbreviations:* U2, U-2 OS; Ibn, ibandronate; MG, MG-63 osteosarcoma cells; PC, PC-3 prostate cancer cells; MDA, MDA-MB-231 breast cancer cells; Sim, simvastatin; A2780, A2780 ovarian cancer cell line; C75, inhibitor of fatty acid synthase.

a All data were derived from Affymetrix ST1.0 expression microarrays, which were used for analysis of mRNA from cell lines after 3 days of treatment with the following drugs: 150 μM ibandronate, 32 μM simvastatin, 27 μM C75). Expression levels are in relation to the set of standard genes in the microarrays; ^∗^ = fold downregulation and † = fold upregulation.

**Table 2:** Down-regulation of HDACs by inhibitors of the mevalonic acid pathway^a^

Gene expression	U2-Ibn	MG-Ibn	PC-Ibn	MDA-Ibn	U2-Sim	MG-Sim	PC-Sim	MDA-Sim	HMC-Sim
*HDAC1* basal expression	10.9	10.2	12.2	10.0	10.5	10.2	11.0	10.0	10.2
*HDAC1* treated expression	10.8	9.9	11.9	9.9	10.7	10.1	10.1	9.7	9.4
***HDAC1* fold expression**	**−1.10^∗^**	**−1.28^∗^**	**−1.21^∗^**	**−1.14^∗^**	**1.11†**	**−1.07^∗^**	**−1.85^∗^**	**−1.31^∗^**	**−1.69^∗^**
*HDAC2* basal expression	10.5	4.6	10.6	8.4	10.5	4.6	8.4	4.3	7.9
*HDAC2* treated expression	10.0	4.5	10.0	8.3	10.4	4.6	8.0	4.5	7.0
***HDAC2* fold expression**	**−1.38^∗^**	**−1.07^∗^**	**−1.44^∗^**	**−1.14^∗^**	**−1.06^∗^**	**1.04†**	**−1.30^∗^**	**1.19†**	**−1.84^∗^**
*HDAC3* basal expression	10.7	9.8	10.9	9.9	10.4	9.8	10.0	9.9	9.8
*HDAC3* treated expression	10.2	9.4	10.6	10.1	10.6	9.6	9.6	9.9	9.3
***HDAC3* fold expression**	**−1.40^∗^**	**−1.33^∗^**	**−1.26^∗^**	**1.09†**	**1.13†**	**−1.20^∗^**	**−1.30^∗^**	**−1.00**	**−1.26^∗^**
*HDAC7* basal expression	9.1	9.3	8.8	9.1	9.1	9.3	9.3	9.1	9.1
*HDAC7* treated expression	8.8	9.2	8.6	9.2	9.0	9.5	8.9	9.0	9.0
***HDAC7* fold expression**	**−1.28^∗^**	**−1.13^∗^**	**−1.14^∗^**	**1.03†**	**−1.09^∗^**	**1.08†**	**−1.34^∗^**	**−1.08^∗^**	**−1.08^∗^**
*HDAC8* basal expression	9.7	8.1	9.8	8.0	9.7	8.1	8.1	8.0	8.7
*HDAC8* treated expression	9.1	7.4	9.6	8.1	9.7	7.8	8.1	7.7	8.7
***HDAC8* fold expression**	**−1.49^∗^**	**−1.68^∗^**	**−1.14^∗^**	**1.03†**	**−1.02**	**−1.25^∗^**	**−1.01**	**−1.28^∗^**	**−1.05^∗^**

*Abbreviations:* HMC, HMC1.1 mast cell line; U2, U-2 OS; MG, MG-63 osteosarcoma cells; MDA, MDA-MB-231 breast cancer cells; PC, PC-3 prostate cancer cells; HMC, HMC1.1 mast cell line; Ibn, ibandronate; Sim, simvastatin; C75, inhibitor of fatty acid synthase.

a All data are derived from Affymetrix ST1.0 expression microarrays, which were used for analysis of mRNA from cell lines after 3 days treatment with respective drugs (150µM ibandronate, 32µM simvastatin, 27µM C75). Expression levels are in relation to the set of standard genes in the microarrays; ^∗^ = fold downregulation and † = fold upregulation.

**Table 3:** Down-regulation of microRNA MIR-34A and up-regulation of microRNA MIR-612 by simvastatin^a^

Gene expression	MG-Sim	PC-Sim	MDA-Sim	HMC-Sim
MIR-34A basal expression	6.3	7.7	6.3	6.4
MIR-34A treated expression	6.1	7.5	6.0	6.4
**MIR-34A fold expression**	**−1.10^∗^**	**−1.16^∗^**	**−1.24^∗^**	**−1.01**
MIR-612 basal expression	7.1	5.9	8.1	7.9
MIR-612 treated expression	7.9	6.6	9.0	9.4
**MIR-612 fold expression**	**1.65†**	**1.65†**	**1.82†**	**2.88†**

*Abbreviations:* U2, U-2 OS; MG, MG-63 osteosarcoma cells; MDA, MDA-MB-231 breast cancer cells; PC, PC-3 prostate cancer cells; HMC, HMC1.1 mast cell line; Ibn, ibandronate; Sim, simvastatin; C75, inhibitor of fatty acid synthase.

a All data are derived from Affymetrix ST1.0 expression microarrays, which were used for analysis of mRNA from cell lines after 3 days treatment with respective drugs (150µM ibandronate, 32µM simvastatin, 27µM C75). Expression levels are in relation to the set of standard genes in the microarrays; ^∗^ = fold downregulation and † = fold upregulation.

**Table 4:** Effect of ibandronate and simvastatin on key enzymes of OCM, namely *DHFR* and *TYMS*^a^

Gene expression	U2-Ibn	MG-Ibn	PC-Ibn	MDA-Ibn	U2-Sim	MG-Sim	PC-Sim	MDA-Sim	A2780-C75
*DHFR* basal expression	10.3	7.9	10.4	7.5	10.2	7.9	7.6	7.5	9.9
*DHFR* treated expression	10.0	7.7	9.2	7.3	10.0	7.7	8.0	6.5	9.4
***DHFR* fold expression**	**−1.16^∗^**	**−1.11^∗^**	**−2.25^∗^**	**−1.14^∗^**	**−1.10^∗^**	**−1.17^∗^**	**1.32†**	**−2.03^∗^**	**−1.37^∗^**
*TYMS* basal expression	11.9	11.1	11.5	10.7	11.9	11.1	11.2	10.7	10.7
*TYMS* treated expression	11.9	10.9	9.8	10.4	11.8	11.1	8.2	7.1	9.6
***TYMS* fold expression**	**−1.05^∗^**	**−1.21^∗^**	**−3.18^∗^**	**−1.23^∗^**	**−1.07^∗^**	**1.00**	**−7.82^∗^**	**−12.40^∗^**	**−2.12^∗^**

*Abbreviations:* U2, U-2 OS; MG, MG-63 osteosarcoma cells; MDA, MDA-MB-231 breast cancer cells; PC, PC-3 prostate cancer cells; A2780, A2780 ovarian cancer cell line; Ibn, ibandronate; Sim, simvastatin; C75, inhibitor of fatty acid synthase.

a All data are derived from Affymetrix ST1.0 expression microarrays, which were used for analysis of mRNA from cell lines after 3 days treatment with respective drugs (150µM ibandronate, 32µM simvastatin, 27µM C75). Expression levels are in relation to the set of standard genes in the microarrays; ^∗^ = fold downregulation and † = fold upregulation.

**Table 5:** Expression of *LOX* was stimulated both by simvastatin and ibandronate^a^

Gene expression	U2-Ibn	MG-Ibn	PC-Ibn	MDA-Ibn	U2-Sim	MG-Sim	PC-Sim	MDA-Sim
*LOX* basal expression	9.3	9.3	8.3	10.2	8.9	9.3	7.9	10.2
*LOX* treated expression	10.0	10.0	9.8	10.3	9.5	9.6	9.9	10.6
***LOX* fold expression**	**1.60†**	**1.67†**	**2.90†**	**1.09†**	**1.50†**	**1.22†**	**4.19†**	**1.39†**

*Abbreviations:* U2, U-2 OS; MG, MG-63 osteosarcoma cells; MDA, MDA-MB-231 breast cancer cells; PC, PC-3 prostate cancer cells; Ibn, ibandronate; Sim, simvastatin; C75, inhibitor of fatty acid synthase.

a All data are derived from Affymetrix ST1.0 expression microarrays, which were used for analysis of mRNA from cell lines after 3 days treatment with respective drugs (150µM ibandronate, 32µM simvastatin, 27µM C75). Expression levels are in relation to the set of standard genes in the microarrays; ^∗^ = fold downregulation and † = fold upregulation.

**Table 6:** Expression of *FASN* was regulated both by simvastatin and ibandronate^a^

Gene expression	U2-Ibn	MG-Ibn	PC-Ibn	MDA-Ibn	U2-Sim	MG-Sim	PC-Sim	MDA-Sim
*FASN* basal expression	10.5	10.9	10.3	9.6	10.3	10.9	10.4	9.6
*FASN* treated expression	9.8	10.9	10.1	9.6	10.2	11.1	8.8	9.5
***FASN* fold expression**	**−1.63^∗^**	**−1.03**	**−1.17^∗^**	**1.02†**	**−1.09^∗^**	**1.16†**	**−3.03^∗^**	**−1.04**

*Abbreviations:* U2, U-2 OS; MG, MG-63 osteosarcoma cells; MDA, MDA-MB-231 breast cancer cells; PC, PC-3 prostate cancer cells; Ibn, ibandronate; Sim, simvastatin; C75, inhibitor of fatty acid synthase.

a All data are derived from Affymetrix ST1.0 expression microarrays, which were used for analysis of mRNA from cell lines after 3 days treatment with respective drugs (150µM ibandronate, 32µM simvastatin, 27µM C75). Expression levels are in relation to the set of standard genes in the microarrays; ^∗^ = fold downregulation and † = fold upregulation.
